# Firearm ownership among American veterans: findings from the 2015 National Firearm Survey

**DOI:** 10.1186/s40621-017-0130-y

**Published:** 2017-12-19

**Authors:** Emily C. Cleveland, Deborah Azrael, Joseph A. Simonetti, Matthew Miller

**Affiliations:** 1 Massachusetts General Hospital, Department of Emergency Medicine, Massachusetts General Hospital Massachusetts General Hospital, 5 Emerson Place, Suite 101, Boston, MA 02114 USA; 2 Harvard Injury Control Research Center, Harvard School of Public Health, Boston, MA USA; 3 Rocky Mountain Mental Illness Research, Education and Clinical Center, Veterans Health Administration, Denver, CO USA; 4 Northeastern University, Bouvé College of Health Sciences, Boston, MA USA

## Abstract

**Background:**

While the majority of veteran suicides involve firearms, no contemporary data describing firearm ownership among US veterans are available. This study uses survey data to describe the prevalence of firearm ownership among a nationally representative sample of veterans, as well as veterans’ reasons for firearm ownership.

**Methods:**

A cross-sectional, nationally representative web-based survey conducted in 2015.

**Results:**

Nearly half of all veterans own one or more firearms (44.9%, 95% CI 41.3–48.6%), with male veterans more commonly owning firearms than do female veterans (47.2%, 95% CI 43.4–51.0% versus 24.4%, 95%CI 15.6–36.1%). Most veteran firearm owners own both handguns and long guns (56.5%, 95% CI 51.1–61.8%); a majority cite protection as a primary reason for firearm ownership (63.1%, 95% CI 58.2–67.8%).

**Conclusions:**

The current study is the first to provide detailed, nationally representative information about firearm ownership among U.S. veterans. Better understanding firearm ownership among veterans can usefully inform ongoing suicide prevention efforts aiming to facilitate lethal means safety among vulnerable veterans during at-risk periods.

**Electronic supplementary material:**

The online version of this article (10.1186/s40621-017-0130-y) contains supplementary material, which is available to authorized users.

## Background

Veterans of the United States (U.S.) Armed Forces accounted for nearly 20% of U.S. adult suicides in 2014 (VA Suicide Prevention Program, 2016). A salient characteristic of veteran suicides is that the great majority involve guns (e.g., 66% of male veteran suicides involve firearms, compared with 56% of US males generally) (VA Suicide Prevention Program, 2016).

Surprisingly, given this observation, no contemporary, peer-reviewed data describing firearm ownership among U.S. veterans are available. Instead, estimates of firearm ownership among U.S. veterans come from studies of small samples of veterans receiving care from the Veterans Health Administration (VHA) (Freeman et al., 1994; Freeman et al., 2003; Freeman & Roca, 2001; Smith et al., 2015; Heinz et al., 2016; Kellermann et al., 1992; Miller et al., 2006; Miller et al., 2007; Wiebe, 2003), and from unpublished nationally representative survey data that are over a decade old: the 2004 Behavioral Risk Factor Surveillance System (BRFSS) (Center for Disease Control and Prevention (CDC), 2004). Data from the public access 2004 BRFSS indicate that veterans are more likely to report living in homes with firearms (49%) than are nonveterans overall (31%), or nonveteran males (36%).

The dearth of information about firearm exposure among veterans is striking given the high priority and scholarly attention accorded to preventing veteran suicide (McCarthy et al., 2015; Hersher, 2017; VA Department of Office of Public and Intergovernmental Affairs, 2017), and the well-established link between firearm ownership and suicide (Kellermann et al., 1992; Wiebe, 2003; Miller & Hemenway, 1999; Anglemyer et al., 2014). The current study addresses this knowledge gap by presenting detailed, nationally representative information about the type, number, distribution, use, and reasons for firearm ownership among veterans. Better understanding firearm exposure and ownership among veterans can usefully inform ongoing suicide prevention efforts aimed at reducing access to firearms for vulnerable veterans during at-risk periods – one of two suicide prevention strategies generally considered to have the strongest empirical foundation (Mann et al., 2005; Azrael & Miller, 2016; Barber & Miller, 2014).

## Methods

### Sampling and design

The National Firearm Survey (NFS) was approved by the Institutional Review Board at Northeastern University. The NFS is a cross-sectional, web-based survey conducted by the survey research firm Growth for Knowledge (GfK) in January 2015. The sample was drawn from Knowledge Panel, an online sampling frame of approximately 55,000 U.S. adults selected using address-based sampling methods maintained by GfK. Participants are selected on an ongoing basis using equal probability sampling. For the purposes of this study, veterans and firearm owners were oversampled to ensure reliable national estimates when conducting pre-planned subpopulation analyses. Active duty military personnel were excluded from participation. Of the 7318 panel members invited to complete the survey, 4165 started and 3949 completed the survey (response rate 54.6%, participation rate 94.8%). Study-specific analytic weights provided by GfK incorporate base weights for Knowledge Panel that adjust the sample to the most recent U.S. Census Current Population Survey (2010) (GfK, 2012), and post-stratification weights that adjust for survey non-response, under- or over-coverage imposed by the study-specific sample design, and benchmark demographic distributions based on the 2014 veteran supplemental survey of the Current Population Survey (GfK, 2012) and the 2014 American Community Survey (National Center for Veterans Analysis and Statistics, 2016). Further detail regarding the adjustment of final weights for the NFS has been previously published (Miller et al., 2017; Azrael et al., 2017).

Invitations to participate in the study were sent by e-mail. One reminder e-mail was sent to non-responders three days later. GfK has a modest point-based incentive program through which participants accrue points for completing surveys and can later redeem them for cash, merchandise, or participation in sweepstakes.

### Measures

Survey items assessed respondents’ sociodemographic characteristics, political views, history of military service in the U.S. Armed Forces and, for those who served, time period of service, and service branch. Veterans were asked about their use of health care services in the last 12 months and categorized by whether they received no, some, or all of that care from the Veterans Health Administration (VHA). We describe veterans discharged from the military in 2002 or later as “recent veterans”. This group encompasses former military personnel who served in the era of Operations Iraqi Freedom, Enduring Freedom, and New Dawn (OIF/OEF/OND).

Firearm ownership status was assessed first by asking “Do you or does anyone you live with currently own any type of gun?” If the answer was affirmative, the question “Do you personally own a gun?” followed. Gun owners were asked about the number and type of gun(s) they own (handgun, long gun, or other gun, as well as sub-types of handguns including pistols and revolvers). Firearm owners were asked whether they owned a gun primarily for protection. In addition, for each type of gun that they owned, they were asked to indicate their reasons for ownership (i.e. why they own their handgun(s), why they own their long gun(s)).

All analyses were conducted using STATA 14.1 (StataCorp, College Station, TX, USA).

## Results

A total of 1044 veterans of the US Armed Forces completed the 2015 National Firearm Survey and were included in this study. The overall survey completion rate was 54.6%. Further details regarding the overall study population have been previously published (Azrael et al., 2017). After accounting for survey weights, this established a nationally representative group of 384 veterans. The mean age of veterans was 60.4 years, 90.1% were male, and 78.4% were non-Hispanic white. See Additional file 1: Table S1 for additional sociodemographic and military service characteristics of the veteran study population.

In comparisons of veterans with non-veterans (Table 1), veterans own firearms more commonly (44.9%, 95% confidence interval [CI] 41.3–48.6%) than do non-veterans (20%, 95% CI 18.3–21.6%) in the general population (Azrael et al., 2017). This is true for both men and women. Among gun owners, however, male veterans and male non-veterans look broadly similar. For example, slightly more than half of all male gun owners, regardless of veteran status, own both handguns and long guns. Among women gun owners, however, non-veterans are more likely than are veterans to own only handguns (42%, 95% CI 36.9–47.3 versus 17.0%, 95%CI 6.7–37%), while veteran women more often own multiple types of firearms (67.0%, 95% CI 45.5–83.1%) than do non-veteran women (42.9%, 95% CI 37.8–48.1%).

**Table 1:** Firearm ownership among veteran versus non-veterans

Among all respondents			Among gun-owning respondents					
Owns any gun			Owns only handgun(s)		Owns only long gun(s)		Owns multiple types	
	%	95% CI:	%	95% CI:	%	95% CI:	%	95% CI:
Veterans (weighted *n* = 384)	44.9	(41.3, 48.6)	22.8	(18.2, 28.0)	20.7	(16.7, 25.4)	56.5	(51.1, 61.8)
Male (*n* = 346)	47.2	(43.4, 51.0)	23.1	(18.4, 28.6)	21.0	(16.8, 25.9)	55.9	(50.3, 61.4)
Female (*n* = 38)	24.4	(15.6, 36.1)	17.0	(6.7, 37.0)	16.0	(6.1, 35.8)	67.0	(45.5, 83.1)
Non-veterans (weighted *n* = 3565)	20.0	(18.3, 21.6)	27.5	(24.8, 30.3)	22.8	(20.4, 25.4)	49.7	(46.7, 52.7)
Male (*n* = 1563)	30.3	(27.0, 33.8)	20.2	(17.3, 23.5)	26.7	(23.6, 30.1)	53.1	(49.4, 56.7)
Female (*n* = 2002)	11.8	(10.4, 13.4)	42.0	(36.9, 47.3)	15.1	(11.7, 19.3)	42.9	(37.8, 48.1)

### Characteristics of veterans by household and personal firearm ownership status:

Male veterans more commonly own a firearm than do female veterans (47.2%, 95% CI 43.4–51.0% versus 24.4%, 95% CI 15.8–35.8%, respectively) (Table 2). Among all veterans, 3 % (95% CI 2.0–5.5%) do not personally own a firearm but live in a household with a gun. However, a greater proportion of female veterans live in a household with a firearm but are not themselves the owner (14.4%, 95% CI 5.5–32.5% among females versus 2.2%, 95% CI 1.3–3.5% among males). Approximately half of male veterans and 60% of female veterans live in homes that do not contain firearms (50.7%, 95% CI 46.8–54.5% and 61.2%, 46.6–74.0%, respectively). The proportion of veterans who own guns increases with age (26.5%, 95% CI 13.8–44.9% for age 18–29 years, up to 51.2%, 95% CI 46.6–55.8% for age 60 and older).

**Table 2:** Demographic characteristics of veterans in the 2015 National Firearm Survey, by firearm ownership

	Firearm owner % (95% CI)	Non-owner, lives with firearm owner % (95% CI)	Non-owner, does not live with firearm owner % (95% CI)
All veterans (weighted n = 384)	44.9 (41.3, 48.6)	3.4 (2.0, 5.5)	51.7 (48.0, 55.4)
Age, years			
18–29 (*n* = 19)	26.5 (13.8, 44.9)	1.1 (0.1,7.4)	72.4 (54.0, 85.5)
30–44 (*n* = 51)	28.2 (19.6, 38.8)	7.9 (2.2, 24.9)	63.9 (51.6, 74.6)
45–59 (*n* = 99)	43.4 (36.3, 50.8)	3.7 (1.9, 7.0)	52.9 (45.5, 60.3)
60+ (*n* = 215)	51.2 (46.6, 55.8)	2.4 (1.3, 4.3)	46.4 (41.9, 51.0)
Sex			
Male (*n* = 346)	47.2 (43.4, 51.0)	2.2 (1.3, 3.5)	50.7 (46.8, 54.5)
Female (*n* = 38)	24.4 (15.8, 35.8)	14.4 (5.5, 32.5)	61.2 (46.6, 74.0)
Race/Ethnicity			
Non-Hispanic White (*n* = 301)	47.8 (43.8, 51.8)	1.9 (1.2, 3.1)	50.3 (46.3, 54.3)
Non-Hispanic Black (*n* = 44)	36.2 (23.4, 51.4)	10.5 (3.2, 28.9)	53.4 (38.7, 67.5)
Non-Hispanic, other (n = 15)	38.9 (22.6, 58.2)	10.3 (3.1, 29.1)	50.8 (32.1, 69.3)
Hispanic (*n* = 24)	29.2 (19.2, 41.7)	4.3 (1.2, 14.1)	66.5 (53.7, 77.3)
Marital Status			
Married / Partnered (*n* = 276)	47.9 (43.7, 52.2)	3.9 (2.2, 6.8)	48.2 (43.9, 52.5)
Widowed / Separated (*n* = 72)	45.7 (36.8, 55.0)	2.1 (0.6, 7.7)	52.1 (42.9, 61.1)
Never married (*n* = 35)	19.7 (11.2, 32.3)	1.4 (0.4, 5.7)	78.9 (66.6, 87.5)
Children under 18 years in household			
No (*n* = 64)	46.9 (42.9, 51.0)	2.4 (1.5, 3.9)	50.7 (46.6, 54.7)
Yes (*n* = 320)	34.9 (27.2, 43.5)	8.3 (3.1, 20.6)	56.8 (47.2, 65.9)
Grew up with a gun in the household			
Yes (*n* = 233)	55.8 (51.1,60.3)	3.5 (1.8,6.8)	40.7 (36.2,45.4)
No (*n* = 128)	29.0 (23.3,35.4)	3.2 (1.4,7.1)	67.9 (61.3,73.8)
Don’t know/refused (n = 23)	24.3 (13.2,40.4)	2.8 (0.6,11.5)	73.0 (56.5,84.9)
Education			
Less than high school (*n* = 12)	40.3 (22.6, 60.8)	1.6 (0.2, 10.9)	58.1 (38.7, 75.4)
High school / some college (*n* = 266)	46.0 (41.5, 50.5)	2.6 (1.6, 4.2)	51.5 (46.9, 56.0)
Bachelor’s degree or more (*n* = 106)	42.9 (36.5, 49.5)	5.6 (2.2, 13.4)	51.5 (44.7, 58.3)
Community type			
Rural (*n* = 107)	59.3 (51.7, 66.4)	4.1 (1.9, 8.9)	36.6 (29.6, 44.2)
Urban (*n* = 88)	35.3 (28.1, 43.2)	2.2 (0.7, 6.7)	62.5 (54.5, 69.8)
Suburban (*n* = 187)	41.6 (36.9, 46.5)	3.5 (1.6, 7.4)	54.9 (49.9, 59.8)
Political views			
Liberal (*n* = 54)	39.0 (30.4,48.3)	1.5 (0.4,5.1)	59.6 (50.2,68.3)
Moderate (*n* = 157)	41.4 (36.1,47.0)	5.8 (3.1,10.5)	52.8 (47.1,58.4)
Conservative (*n* = 166)	50.5 (44.6,56.3)	1.8 (0.6,5.1)	47.7 (41.9,53.6)
U.S. Region			
Northeast (*n* = 57)	42.1 (33.3, 51.3)	1.3 (0.4, 4.1)	56.6 (47.4, 65.5)
Midwest (*n* = 86)	38.6 (31.8, 46.0)	4.5 (2.2, 8.8)	56.9 (49.4, 64.1)
South (*n* = 152)	52.3 (46.1, 58.5)	4.3 (1.8, 9.7)	43.4 (37.4, 49.7)
West (*n* = 88)	40.2 (33.4, 47.5)	2.1 (0.8, 5.8)	57.7 (50.3, 64.7)
Service Branch			
Army (*n* = 179)	45.7 (40.3, 51.3)	3.7 (1.6, 8.1)	50.6 (45.0, 56.2)
Navy (*n* = 78)	44.6 (36.9, 52.4)	1.7 (0.7, 4.1)	53.8 (45.9, 61.5)
Air Force (*n* = 75)	45.8 (38.6, 53.2)	5.6 (2.4, 12.5)	48.6 (41.1, 56.2)
Marine Corps (*n* = 37)	48.4 (35.1, 61.9)	3.3 (1.0, 10.6)	48.3 (35.0, 61.9)
Coast Guard (n = 5)	52.8 (20.8, 82.6)	(none)	47.2 (17.4, 79.2)
Recent military service^a^			
No (*n* = 329)	45.5 (41.7, 49.4)	2.5 (1.6, 3.8)	52.0 (48.1, 56.0)
Yes (*n* = 47)	40.5 (30.3, 51.6)	10.5 (3.4, 28.0)	49.0 (37.5, 60.6)
Use of VHA services in past year^b^			
None (*n* = 295)	44.4 (40.3, 48.7)	3.4 (1.8, 6.0)	52.2 (47.9, 56.5)
For some care (*n* = 86)	50.5 (40.0, 61.0)	0.7 (0.1, 4.6)	48.8 (38.4, 59.4)
For all care (*n* = 43)	42.9 (32.7, 53.9)	6.2 (2.4, 15.0)	50.9 (40.0, 61.7)

*VHA* Veterans Health Administration, *CI* confidence interval

^a^Recent service includes Veterans whose last year of active duty was 2002 or later

^b^Indicates utilization of any VHA healthcare services

All n’s are reported based on survey weights

Veterans who have never been married less commonly own a firearm (19.7%, 95% CI 11.2–32.3%) than those who are currently or were previously married (47.8%, 95% CI 43.8–51.8 for married/partnered veterans; 45.7%, 95% CI 36.8–55.0% for widowed/separated veterans). Veterans with children under the age of 18 in their household less commonly own a firearm than those without children (34.9%, 95% CI 27.2–43.5% versus 46.9%, 95% CI 42.9–51.0%, respectively). Veterans who grew up without a firearm in their household also less often own a gun than those who grew up with a gun in the home (29.0%, 95% CI 23.3–35.4% versus 55.8%, 95% CI 51.1–60.3%).

More than half of veterans living in Southern states own a firearm (52.3%, 95%CI 46.1–58.5%). In contrast, approximately two in five veterans living in other regions own firearms. Indeed, veterans residing in the South account for nearly half of all veteran firearm owners (46.2%, 95%CI 41.1–51.3%, data not shown). Prevalence of firearm ownership does not differ substantially by service branch, service era, or use of VHA health care services. When the analysis was restricted to male veterans only, no important differences were identified (see Additional file 1: Table S1).

### Gunstock among veteran gun owners

Most veterans who own guns have multiple types (56.8%, 95% CI 51.4–62.1%), while 22.6% report owning only handguns (95% CI 18.1–27.8%) and 20.6% own only long guns (95% CI 16.6–25.2%, see Table 3). This pattern held across most demographic categories examined, including age, sex, educational achievement, presence of children in the household, political views, branch of service, recency of military service, and use of VHA services. A greater proportion of non-Hispanic Black than non-Hispanic white veteran gun owners own only a handgun (63.8%, 95% CI 38.5–83.3% versus 17.6%, 95% CI 14.0–21.9%). Non-Hispanic whites more commonly own multiple types of firearms (60.0%, 95% CI 54.5–65.3%, versus 24.6%, 95% CI 9.9–49.1). Veterans living in urban areas more commonly own only a handgun than do veterans living in rural areas (39.5%, 95% CI 26.5–54.3% versus 16.3%, 95% CI 9.6–26.4%).

**Table 3:** Demographic characteristics of veteran gun owners by firearm type

	Owns only handgun(s)		Owns only long gun(s)		Owns multiple types	
	%	95% CI	%	95% CI:	%	95% CI:
All veteran gun owners (*n* = 159)	22.8	(18.2, 28.0)	20.7	(16.7, 25.4)	56.5	(51.1, 61.8)
Age						
18–29 (n = 4)	23.2	(6.5, 60.0)	23.4	(5.9, 59.8)	53.4	(23.3, 81.3)
30–44 (*n* = 13)	18.0	(7.5, 48.3)	22.2	(8.0, 48.3)	59.8	(38.7, 77.8)
45–59 (*n* = 40)	25.7	(16.2, 38.2)	18.6	(11.7, 28.1)	55.8	(44.4, 66.6)
60+ (*n* = 103)	22.2	(16.9, 28.6)	21.3	(16.5, 26.9)	56.6	(50.0, 62.9)
Sex						
Male (*n* = 152)	23.1	(18.4, 28.6)	21.0	(16.8, 25.8)	55.9	(50.4, 61.4)
Female (*n* = 8)	17.0	(7.1, 35.6)	16.0	(6.5, 34.4)	67.0	(46.8, 82.4)
Race						
Non-Hispanic white (*n* = 133)	17.7	(14.1, 22.0)	22.3	(17.8, 27.6)	60.0	(54.5, 65.3)
Non-Hispanic Black (*n* = 14)	67.1	(42.7, 84.8)	8.4	(2.8, 22.9)	24.6	(9.9, 49.1)
Non-Hispanic other (*n* = 6)	23.6	(5.6, 61.6)	17.7	(5.6, 43.5)	58.8	(30.9, 82.0)
Hispanic (*n* = 7)	31.4	(15.3, 53.6)	16.7	(6.5, 36.5)	51.9	(32.1, 71.1)
Marital status						
Married/Partnered (*n* = 124)	22.0	(17.4, 27.4)	20.6	(16.4, 25.5)	57.4	(51.7, 62.9)
Widowed/Separated (*n* = 30)	22.7	(11.3, 40.5)	22.1	(11.9, 37.5)	55.1	(39.8, 69.6)
Never married (*n* = 6)	39.1	(15.3, 69.6)	15.3	(4.0, 44.0)	45.6	(18.0, 76.2)
Child under 18yo in the household						
Yes (*n* = 22)	25.7	(16.1, 38.4)	23.2	(14.0, 35.9)	51.1	(38.5, 63.6)
No (*n* = 138)	22.3	(17.4, 28.1)	20.3	(16.0, 25.4)	57.4	(51.4, 63.1)
Grew up with a firearm in the home						
Yes (*n* = 118)	16.4	(12.0, 22.1)	20.3	(16.2, 25.1)	63.3	(57.3, 68.8)
No (*n* = 35)	40.2	(28.4, 53.2)	23.5	(13.5, 37.6)	36.4	(25.8, 48.5)
Don’t know/refused (*n* = 5)	47.5	(22.7, 73.5)	12.1	(2.5, 42.5)	40.4	(18.0, 67.6)
Education						
Less than High School (*n* = 4)	25.2	(7.7, 57.6)	10.0	(2.3, 34.3)	64.9	(34.9, 86.4)
High school/some college (*n* = 112)	24.1	(18.4, 30.9)	18.8	(14.4, 24.1)	57.1	(50.5, 63.6)
BA or more (*n* = 42)	19.0	(12.7, 27.3)	27.0	(18.7, 37.2)	54.1	(44.7, 63.2)
Community						
Urban (n = 29)	38.2	(25.6, 52.6)	15.7	(9.4, 25.1)	46.1	(33.8, 59.0)
Suburban (*n* = 71)	21.3	(16.1, 27.6)	19.1	(14.2, 25.2)	59.6	(52.6, 66.2)
Rural (*n* = 58)	16.5	(9.8, 26.6)	25.4	(17.6, 35.1)	58.1	(48.0, 67.6)
Political views						
Liberal (*n* = 19)	22.8	(13.2, 36.5)	21.4	(12.3, 34.6)	55.8	(41.8, 69.0)
Moderate (*n* = 58)	28.5	(20.8, 37.6)	18.1	(12.7, 25.1)	53.5	(44.9, 61.8)
Conservative (*n* = 80)	18.9	(12.9, 26.7)	22.7	(16.7, 30.2)	58.4	(50.3, 66.1)
Geographic region						
Northeast (*n* = 21)	20.9	(11.3, 35.3)	27.6	(17.3, 41.1)	51.5	(37.5, 65.2)
Midwest (*n* = 32)	14.9	(9.3, 23.1)	27.8	(18.7, 39.2)	57.3	(46.2, 67.6)
South (*n* = 74)	28.7	(21.0, 37.9)	15.8	(10.5, 23.0)	55.5	(46.9, 63.9)
West (*n* = 32)	18.0	(11.6, 26.7)	20.6	(12.9, 31.2)	61.5	(50.8, 71.2)
Branch of service						
Army (*n* = 77)	22.7	(16.2, 30.8)	16.9	(11.9, 23.4)	60.4	(52.2, 68.1)
Navy (*n* = 30)	26.3	(15.4, 41.0)	21.1	(13.5, 31.4)	52.6	(40.4, 64.5)
Air Force (*n* = 30)	21.4	(13.9, 31.4)	20.7	(13.8, 29.9)	57.9	(47.8, 67.4)
Marine Corps (*n* = 14)	21.6	(8.9, 43.7)	35.3	(17.0, 59.3)	43.1	(25.0, 63.1)
Coast Guard (*n* = 3)	8.9	(0.1, 61.6)	35.0	(3.4, 89.2)	56.0	(10.8, 93.1)
Recent military service						
Yes (*n* = 18)	27.3	(15.4, 43.6)	11.8	(5.3, 24.3)	60.9	(45.4, 74.4)
No (*n* = 140)	22.3	(17.5, 28.0)	21.4	(17.1, 26.5)	56.3	(50.5, 62.0)
Use VHA services						
Yes (*n* = 36)	24.5	(16.7, 34.4)	19.6	(11.5, 31.3)	55.9	(44.9, 66.4)
No (*n* = 122)	22.3	(17.0, 28.7)	21.1	(16.7, 26.4)	56.6	(50.3, 62.7)

VHA, Veterans Health Administration. “Use VHA services” indicates a veteran who reports receiving any healthcare through the VHA in the last 12 months

Recent veterans include those whose last year of active duty was 2002 or later

All n’s are reported based on weighted values

Rifles and pistols are the most commonly owned gun types, followed by shotguns and revolvers. These proportions do not differ substantially by gender, region, VHA healthcare use, or by recency of military service (see Table 4). Approximately 20% of veteran gun owners own only one gun, and nearly 45% own either one or two firearms (see Table 4). Female veterans on average own fewer firearms than their male counterparts (4.5, 95% CI 2.6–6.4 versus 6.2, 95% CI 4.2–8.1). However, the overall gunstock appears to be more concentrated among female veterans: 45% of female veterans who own any gun possess 6 or more firearms (95%CI 23.8–68.2%, data not shown), compared to 29.8% of males (95% CI 25.4–34.8%, not shown). Among gun owning veterans, there are no substantial regional differences in the number of firearms owned. While the average number of guns owned by veterans in the Northeast and West appears higher than in other regions, these estimates have both wide and overlapping confidence intervals. The number of firearms owned and the distribution of the gunstock does not appear to vary substantially based on use of VHA services or recency of military service. When the analysis was restricted to male veterans only, no important differences were identified (see Additional file 1: Table S2).

**Table 4:** Gunstock among veteran firearm owners

	Average number of guns		Distribution of guns among veterans				Proportion of veterans owning each type of firearm			
	#	95% CI	1 gun (%)	2 guns (%)	3–5 guns (%)	6+ guns (%)	Pistol (%)	Revolver (%)	Rifle (%)	Shotgun (%)
All veterans^a^	6.1	(4.2, 7.9)	22.9	21.1	25.6	30.5	58.3	40.0	60.0	52.4
Males	6.2	(4.2, 8.1)	23.2	20.8	26.3	29.8	58.0	39.8	59.8	52.6
Females	4.5	(2.6, 6.4)	17.6	26.7	10.7	45.0	62.3	44.3	63.2	49.8
Region										
Northeast	8.4	(1.5, 15.2)	23.4	17.2	30.0	29.6	58.2	33.6	63.4	46.3
Midwest	5.2	(3.2, 7.2)	17.7	25.9	22.6	33.9	53.4	29.2	70.5	61.3
South	4.8	(3.9, 5.8)	26.2	19.8	24.2	29.8	62.0	47.4	55.5	52.8
West	8.2	(1.0, 15.4)	19.8	22.2	28.5	29.5	54.3	38.0	58.0	47.4
VHA use										
Any VHA care	5	(3.8, 6.1)	22.2	18.9	28.5	30.4	51.6	45.5	57.5	56.2
No VHA care	6.4	(4.0, 8.8)	23.2	21.8	24.7	30.3	60.2	38.5	60.6	51.1
Recent service^b^										
Yes	4.2	(3.1, 5.4)	24.9	18.5	24.9	31.6	61.5	31.5	52.6	55.3
No	6.4	(4.2, 8.5)	22.4	21.5	25.6	30.5	58.1	41.3	60.9	52.2

^a^among veterans who own any firearm

VHA, Veterans Health Administration. “Any VHA services” indicates a veteran who indicates they received any healthcare through the VHA in the last 12 months

^b^Recent veterans include those whose last year of active duty was 2002 or later

### Reason for firearm ownership among veteran gun owners

Overall, 63.1% of veterans firearm owners report that protection against people is the primary reason for ownership (95% CI 58.2–67.8%) (see Table 5). Responses to this question were analyzed among subgroups of veterans who own only handgun(s) and those who own only long gun(s). When comparing these subgroups, a significantly greater proportion of veterans who own only handgun(s) (75.6%, 95% CI 64.3–84.0%) report protection as their primary reason for doing so, compared to 29.2% of veterans who own only long gun(s) (95% CI 19.1–42.0%).

**Table 5:** Reasons for firearm ownership among veterans, by firearm type

	%	95% CI
Own any gun *primarily* for protection against people	63.1	(58.2, 67.8)
Own primarily for protection, by type of firearm owned		
Protection as *primary* reason, own handgun(s) only	75.6	(64.8, 83.9)
Protection as *primary* reason, own long gun(s) only	29.2	(19.1, 42.0)
Protection as *primary* reason, own multiple gun types	69.5	(63.6, 74.9)
Reasons for handgun ownership (select all that apply)		
Protection against strangers	73.2	(67.9, 78.0)
Protection against people I know	4.9	(3.2, 7.5)
Protection against animals	18.3	(14.6, 22.7)
Protection against unspecified	2.1	(1.0, 4.3)
Hunting	23.2	(18.5, 28.8)
Other sporting use	33.6	(28.5, 39.2)
For a collection	19.8	(15.7, 24.6)
Work	2.5	(1.2, 4.9)
Inherited	1.4	(0.7, 2.8)
Gift	1.1	(0.5, 2.6)
Declaration of right	0.3	(0.0, 2.1)
Other reason	7.9	(5.3, 11.5)
Reasons for long gun ownership (select all that apply)		
Protection against strangers	34.3	(29.1, 39.9)
Protection against people I know	2.5	(1.4, 4.5)
Protection against animals	14.7	(11.4, 18.7)
Protection against unspecified	0.1	(0.0, 0.7)
Hunting	59.7	(53.9, 65.2)
Other sporting use	45.1	(39.5, 50.8)
For a collection	28.0	(23.1, 33.4)
Work	0.2	(0.0, 1.6)
Inherited	2.9	(1.8, 4.9)
Gift	2.1	(0.7, 6.6)
Declaration of right	2.0	(0.9, 4.4)
Other reason	4.1	(2.6, 6.4)

Respondents were also given the opportunity to list multiple reasons why they own either a handgun or a long gun (Table 5). Among veterans who own at least one handgun, protection against strangers is the most commonly selected reason for firearm ownership (73.2%, 95% CI 67.9–78.0%). Other common reasons for handgun ownership include hunting (23.2%, 95% CI 18.5–28.8%), other sporting use (33.6%, 95% CI 28.5–39.2%) and for a collection (19.8%, 95% CI 15.7–24.6%). In contrast, veterans who own at least one long gun indicate protection against strangers as a reason for ownership only 34.3% of the time (95% CI 29.1–39.9%), while hunting (59.7%, 95% CI 53.9–65.2%) and other sporting use (45.1%, 95% CI 39.5–50.8%) are more common reasons for long gun ownership. Over a quarter of veterans indicate that having a long gun as part of their collection is a main reason for ownership (28.0%, 95% CI 23.1–33.4%). Reasons for firearm ownership were not different when analysis was restricted to males only (see Additional file: Table S3).

## Discussion

The current study is the first to examine firearm ownership among a nationally representative sample of veterans of the United States Armed Forces. It is also the first to provide details regarding the characteristics of veteran gun owners, the reasons they own their firearms, and the type, number, and distribution of their firearms. Our study shows that veterans own firearms more commonly (45%) than do non-veterans (20%) in the general population (Azrael et al., 2017) (Table 1). This difference is more pronounced among females than among males, with approximately one in four female veterans owning guns (compared with one in nine non-veteran females), and one in two male veterans owning guns, compared with one in three male non-veterans (see Table 1). As males account for the vast majority of veterans, subgroup analyses restricted to males, as expected, were qualitatively similar to our main findings (see Additional file 1: Tables S1-S3). As the proportion of women in the armed forces continues to rise, further research regarding the prevalence and reasons for firearm ownership among female veterans is warranted.

Although veterans are more likely to own firearms than are non-veterans, in general, firearm-owning veterans are similar to firearm owners in the general population (Azrael et al., 2017): when compared with veterans who do not own firearms, veteran firearm owners are more likely to be men, over age 60, non-Hispanic white, currently or previously married, own firearm for protection, and live in rural areas. With respect to the latter, the higher prevalence of firearm ownership among veterans living in rural communities may help to explain the higher rate of firearm deaths in rural veteran suicides compared to urban veteran suicides (McCarthy et al., 2012). In conjunction with data demonstrating higher prevalence of firearm ownership among veterans in the South, these findings suggest subgroups of veterans among whom firearm safety initiatives may produce the greatest benefit.

In the overall US population, nearly seven in ten gun owners report protection as their primary reason for possessing a firearm (Azrael et al., 2017). Similarly, our study found that nearly two-thirds of veterans who own a firearm list protection as a primary reason for ownership. Much as in the general population (Azrael et al., 2017), reasons for gun ownership differ among veterans based on the type of firearm owned. Long guns are more commonly owned by veterans for hunting or other sporting use than for protection, though over a third of veterans who own long guns report protection as a primary reason for ownership.

Recent data on veteran suicides found that male veterans who use VHA services are 22% more likely to die by suicide than male veterans who do not use the VHA (Office of Suicide Prevention, 2016). That we find a similar proportion of firearm ownership across these two groups suggests that the difference in suicide risk by VHA status is attributable to other factors. Indeed, important differences between veterans using and not using VHA services with respect to suicide risk factors have been well documented. For example, prior studies find that VHA-utilizing veterans are far more likely to have lower socioeconomic status and a higher prevalence of mental health, substance-related, and chronic medical conditions (Hoerster et al., 2012; Nelson et al., 2007). The VHA is a recognized leader in public health approaches to suicide prevention, and has begun training providers in lethal means safety counseling (Mental Illness Research Education and Clinical Centers (MIRECC) - US Department of Veterans Affairs, 2017). The VA also distributes firearm locking devices to those at risk of suicide (Nalpathanchil, 2013). Our findings are informative for VHA, as a basic epidemiologic understanding of firearm ownership and use among veterans is necessary to expand such efforts and tailor them to those most likely to benefit.

Three additional implications of our findings are worth highlighting. First, nearly half of male veterans own a firearm or live in a household with a firearm, suggesting that lethal means interventions must be prepared to address firearm access among a large number of veterans. Second, our overall observation that female veterans are more likely to own firearms than are non-veteran females supports previous findings that female veterans may be more likely than their non-veteran counterparts to die from suicide (VA Suicide Prevention Program, 2016), and when they do, more often use a firearm (Kaplan et al., 2009a; Kaplan et al., 2009b). Third, while female veterans are less likely to own firearms than their male counterparts, lethal means safety interventions among the veteran population must recognize the importance of addressing firearm ‘access’ rather than focusing solely on ‘ownership.’ Limiting interventions only to those who report firearm ownership, for example, will neglect the 14% of female veterans who live in households with firearms but do not consider themselves ‘owners.’

By design, the demographic characteristics of veterans responding to our survey closely mirror the distribution of characteristics among the overall veteran population (National Center for Veterans Analysis and Statistics, 2016) that served as a gold standard for the weights in our survey (shown in Additional file 1: Table S1). Veterans in this study who reported *using* the VHA for some or all of their healthcare services in the preceding year appear demographically similar to previously published data for veterans *enrolled* in VHA healthcare services with regard to age, sex, race, marital status, geographic distribution, and service during the OIE/OIF/OND era (data not shown) (Westat, 2010; Gasper et al., 2015). Though differences between veterans *enrolled* in VHA services (some of whom do not use the VHA) and those *using* VHA services may be important, findings from our survey may nevertheless be informative for the VHA given that veterans engaged in VHA care are those most likely to benefit from its services.

Findings from our survey should be interpreted in light of several limitations. Some gun owners may have chosen not to report their firearm ownership on a survey, particularly if they own firearms illegally. Nevertheless, fewer than 1% of NFS respondents refused to answer the initial question about the presence of a gun in the household, and none refused the subsequent question about whether they personally own a gun. While all data are self-reported, the relatively anonymous nature of the online format mitigates social desirability bias and likely yields more accurate estimates of respondent characteristics than comparable telephone surveys (Chang et al., 2009). Furthermore, self-selection bias in this study is to some extent mitigated by high rates of completion for our survey, and information about panelists who elect not to complete the survey informs us about potential differences between those who chose to participate and those who declined. The completion rate for the 2015 NFS was 54.6% overall, and 60.9% for veterans, markedly higher than those of typical nonprobability, opt-in, online surveys, which have completion rates of 2 to 16% (Callegaro & Disogra, 2008). This response rate is also substantially higher than those of previous national injury surveys that included questions about firearm ownership (Centers for Disease Control and Prevention, 2007; Hepburn et al., 2007), and comparable to those from other surveys conducted by GfK (Larrimore et al., 2015). The incentives offered by GfK are modest, but may have introduced selection bias into this study.

## Conclusion

The current study presents the most comprehensive data available regarding firearm ownership patterns among veterans of the United States Armed Forces. Our finding that a larger proportion of male and female veterans own firearms than do male and female non-veterans is consistent with the fact that the proportion of suicides involving firearms among veterans is considerably higher than that among non-veterans (Office of Suicide Prevention, 2016; Miller et al., 2012; Miller et al., 2009). Moreover, our finding that approximately half of all veterans personally own firearms underscores the importance of incorporating efforts to reduce firearm access during at-risk periods into all veteran suicide prevention initiatives. Such an approach is recognized as one of the few empirically established strategies to suicide prevention programs in general (Mann et al., 2005), and is also a strategy that is increasingly supported by the VA (Mental Illness Research Education and Clinical Centers (MIRECC) - US Department of Veterans Affairs, 2017; Nalpathanchil, 2013; Coulson, 2016).

## Additional file

Additional file 1: Supplemental Tables – Male subgroup analysis. (DOCX 48 kb)
