# Supplementary material for: Firearm ownership among American veterans: findings from the 2015 National Firearm Survey
Source: Inj Epidemiol. 2017 Dec 19;4:33. doi: 10.1186/s40621-017-0130-y (PMC5735043; doi:10.1186/s40621-017-0130-y)
Supplement: Additional file 1: — Supplemental Tables – Male subgroup analysis. (DOCX 48 kb) [file 40621_2017_130_MOESM1_ESM.docx]

**Additional file 1: Supplemental Tables – Male subgroup analysis**

**Table S1:** **Demographic characteristics of male veterans in the 2015 National Firearm Survey (compare to Tables 2 and 3)**

|  | **All veterans** | | **All male veterans** | | **Male veteran firearm owners** | |
| --- | --- | --- | --- | --- | --- | --- |
|  | **%** | **95%CI** | **%** | **95%CI** | **%** | **CI** |
| All male veterans |  |  |  |  | 47.2 | 43.4-51.0 |
| Age |  |  |  |  |  |  |
| 18-29 (n=11) | 5.0 | 3.3-7.5 | 3.3 | 2.1-5.2 | 33.3 | 15.2-58.2 |
| 30-44 (n=39) | 13.2 | 10.5-16.4 | 11.3 | 8.8-14.3 | 32.3 | 21.9-44.9 |
| 45-59 (n=252) | 25.9 | 22.7-29.4 | 25.1 | 21.8-28.7 | 45 | 37.2-53.1 |
| 60+ (n=662) | 56.0 | 52.1-59.7 | 60.3 | 56.4-64.1 | 51.6 | 46.9-56.3 |
| Sex |  |  |  |  |  |  |
| Male | 90.1 | 87.1-92.5 | n/a |  | 47.2 | 43.4-51.0 |
| Female | 9.9 | 7.5-12.9 | n/a |  | n/a |  |
| Race |  |  |  |  |  |  |
| White | 78.4 | 74.7-81.7 | 79.4 | 75.8-82.7 | 49.5 | 45.3-53.6 |
| Black | 11.6 | 8.8-15.0 | 11 | 8.3-14.4 | 42.2 | 28.0-57.8 |
| Other | 3.8 | 2.6-5.5 | 1.8 | 2.2-5.0 | 39.4 | 31.4-60.9 |
| Hispanic | 6.3 | 4.8-8.2 | 6.3 | 4.7-8.3 | 31.1 | 20.2-44.7 |
| Marital status |  |  |  |  |  |  |
| Married/Partnered | 72.0 | 68.4-75.4 | 73.2 | 69.4-76.6 | 49 | 44.7-53.4 |
| Widowed/Separated | 18.9 | 16.0-22.1 | 18.2 | 15.3-21.6 | 51.4 | 41.6-61.0 |
| Never married | 9.1 | 7.1-11.7 | 8.6 | 6.6-11.2 | 22.6 | 12.8-36.6 |
| Community |  |  |  |  |  |  |
| Urban | 23.0 | 19.9-26.4 | 23.2 | 19.9-26.8 | 36.6 | 29.0-45.0 |
| Suburban | 49.0 | 45.3-52.8 | 49.5 | 45.7-53.3 | 43.7 | 38.8-48.8 |
| Rural | 28.0 | 24.6-31.6 | 27.4 | 24.0-31.0 | 62.2 | 54.7-69.2 |
| Education |  |  |  |  |  |  |
| Less than HS | 3.1 | 2.1-4.5 | 2.9 | 1.9-4.3 | 43.9 | 24.6-65.1 |
| HS/some college | 69.3 | 65.9-72.6 | 69.6 | 66.1-72.9 | 48.1 | 43.4-52.9 |
| BA or more | 27.6 | 24.5-31.0 | 27.5 | 24.3-30.9 | 45.1 | 38.5-51.9 |
| Branch of service |  |  |  |  |  |  |
| Army | 49.2 | 45.4-52.9 | 47.5 | 43.7-51.4 | 47.9 | 42.2-53.6 |
| Navy | 20.4 | 17.7-23.4 | 19.5 | 16.8-22.5 | 48.1 | 40.3-56.1 |
| Air Force | 19.5 | 17.0-22.4 | 17.9 | 15.5-20.6 | 48.6 | 41.3-56.0 |
| Marine Corps | 9.7 | 7.6-12.4 | 9.5 | 7.3-12.3 | 47.7 | 33.9-61.9 |
| Coast Guard | 1.4 | 0.7-2.5 | 1.4 | 0.7-2.6 | 56.3 | 20.2-86.7 |
| Recent military service |  |  |  |  |  |  |
| Yes | 12.5 | 10.2-15.4 | 10.5 | 8.4-13.1 | 48 | 36.5-59.8 |
| No | 87.5 | 84.6-89.9 | 89.5 | 86.9-91.6 | 47.7 | 43.7-51.8 |
| Use VHA services |  |  |  |  |  |  |
| Yes | 22.7 | 19.8-25.9 | 23.3 | 20.2-26.6 | 46.3 | 38.7-54.0 |
| No | 77.3 | 74.1-80.2 | 76.1 | 72.6-79.3 | 47.9 | 43.5-52.2 |
| Political views |  |  |  |  |  |  |
| Liberal | 14.4 | 12.0-17.2 | 14.2 | 11.7-17.0 | 41.2 | 32.0-51.0 |
| Moderate | 41.6 | 37.9-45.3 | 41.1 | 37.4-45.0 | 43.5 | 37.9-49.3 |
| Conservative | 44.1 | 40.3 47.9 | 44.7 | 40.9-48.6 | 52.3 | 46.2-58.3 |
| Geographic region |  |  |  |  |  |  |
| Northeast | 15.0 | 12.6-17.8 | 15.4 | 12.9-18.4 | 43.8 | 34.5-53.5 |
| Midwest | 22.4 | 19.5-25.6 | 22 | 19.0-25.3 | 39.2 | 31.9-46.9 |
| South | 39.7 | 36.0-43.4 | 39.2 | 35.5-43.1 | 57.1 | 50.8-63.2 |
| West | 23.0 | 20.1-26.3 | 23 | 20.3-26.7 | 40.4 | 33.2-48 |
| Child under 18yo in the household |  |  |  |  |  |  |
| Yes | 16.7 | 14.0-19.8 | 14.2 | 11.7-17.1 | 40.2 | 31.1-50.1 |
| No | 83.4 | 80.3-86.1 | 85.8 | 82.9-88.3 | 48.3 | 44.2-52.5 |
| Grew up with a firearm in the home |  |  |  |  |  |  |
| Yes | 60.7 | 56.9-64.4 | 61.7 | 57.7-65.4 | 57.8 | 53-62.4 |
| No | 33.3 | 29.8-37.0 | 32.7 | 29.1-36.5 | 30.6 | 24.4-37.6 |
| Don't know/refused | 6.1 | 4.2-8.7 | 5.6 | 3.9-8.1 | 27.8 | 15.1-45.3 |

**Table S2: Gunstock among only male veterans (compare to Table 4)**

|  | **Average number of guns owned by male veterans** | | **Distribution of guns among male veterans** | | | | **Proportion of male veterans owning  each type of firearm** | | | |
| --- | --- | --- | --- | --- | --- | --- | --- | --- | --- | --- |
|  | **#** | **95%CI** | **1 gun**  (%) | **2-3 guns**  (%) | **4-5 guns**  (%) | **6+ guns**  (%) | **Pistol**  (%) | **Revolver**  (%) | **Rifle**  (%) | **Shotgun**  (%) |
| Region |  |  |  |  |  |  |  |  |  |  |
| Northeast | 8.6 | (1.6-15.7) | 23.7 | 16.0 | 30.3 | 30.0 | 60.2 | 34.8 | 64.3 | 46.6 |
| Midwest | 5.3 | (3.1-7.5) | 17.8 | 26.6 | 23.0 | 32.5 | 53 | 28.3 | 70.7 | 61 |
| South | 4.8 | (3.8-5.8) | 26.7 | 19.2 | 24.7 | 29.4 | 61.8 | 46.5 | 54.9 | 52.9 |
| West | 8.5 | (0.7-16.3) | 19.1 | 22.7 | 30.4 | 27.8 | 52.1 | 37.9 | 58.3 | 48.3 |
| VHA use |  |  |  |  |  |  |  |  |  |  |
| Any/some | 4.9 | (3.7-6.1) | 21.9 | 19.3 | 30.0 | 28.9 | 50.8 | 45.2 | 57.3 | 55.4 |
| None | 6.6 | (4.0-9.1) | 23.5 | 21.3 | 25.2 | 30.0 | 60.2 | 38.2 | 60.6 | 51.7 |
| Recent service^ |  |  |  |  |  |  |  |  |  |  |
| Yes | 4.4 | (3.1-5.7) | 26.8 | 14.9 | 27.7 | 30.7 | 62.6 | 27.8 | 52.1 | 57.7 |
| No | 6.4 | (4.2-8.6) | 22.5 | 21.6 | 26.1 | 29.8 | 57.8 | 41.4 | 60.7 | 52.1 |

VHA, Veterans Health Administration. “Any VHA services” indicates a veteran who indicates they received any healthcare through the VHA in the last 12 months.

^Recent veterans include those whose last year of active duty was 2002 or later.

**Table S3: Reasons for firearm ownership among male veterans, by firearm type (compare to Table 5)**

|  | % | 95% CI |
| --- | --- | --- |
| Own any gun *primarily* for protection against people | 75.5 | 64.3-84.0 |
| Own primarily for protection, by type of firearm owned |  |  |
| Protection as *primary* reason, own handgun(s) only | 28.6 | 18.2-41.8 |
| Protection as *primary* reason, own long gun(s) only | 71.0 | 65.0-76.4 |
| Protection as *primary* reason, own multiple gun types | 63.8 | 58.7-68.5 |
| Reasons for **handgun** ownership (select all that apply) |  |  |
| Protection against strangers | 73.6 | 68.2-78.4 |
| Protection against people I know | 5 | 3.2-7.7 |
| Protection against animals | 18.3 | 14.5-22.7 |
| Protection against unspecified | 2.2 | 1.1-4.6 |
| Hunting | 23 | 18.2-28.6 |
| Other sporting use | 32.5 | 27.3-38.1 |
| For a collection | 19.9 | 15.7-24.9 |
| Work | 2.6 | 1.3-5.2 |
| Inherited | 1.4 | 0.7-3.0 |
| Gift | 1 | 0.4-2.5 |
| Declaration of right | 1.7 | 0.8-2.2 |
| Other reason | 8.1 | 5.5-11.9 |
| Reasons for **long gun** ownership (select all that apply) |  |  |
| Protection against strangers | 34 | 28.7-39.7 |
| Protection against people I know | 2.6 | 1.5-4.7 |
| Protection against animals | 14.3 | 11.1-18.4 |
| Protection against unspecified | 0.097 | 0.001-0.7 |
| Hunting | 60.5 | 54.6-66.2 |
| Other sporting use | 44.3 | 38.6-50.1 |
| For a collection | 22.8 | 22.9-33.3 |
| Work | 0.2 | 0.03-1.7 |
| Inherited | 2.9 | 1.7-4.9 |
| Gift | 2.2 | 0.7-7.0 |
| Declaration of right | 1.5 | 0.07-3.5 |
| Other reason | 4 | 2.5-6.4 |
